# Microcirculatory, mitochondrial, and histological changes following cerebral ischemia in swine

**DOI:** 10.1186/1471-2202-15-2

**Published:** 2014-01-03

**Authors:** Olga Suchadolskiene, Andrius Pranskunas, Giedre Baliutyte, Vincentas Veikutis, Zilvinas Dambrauskas, Dinas Vaitkaitis, Vilmante Borutaite

**Affiliations:** 1 Department of Disaster Medicine, Lithuanian University of Health Sciences, Eiveniu 4, LT-50161 Kaunas, Lithuania; 2 Department of Intensive Care Medicine, Lithuanian University of Health Sciences, Eiveniu str.2, LT-50009 Kaunas, Lithuania; 3 Institute of Neurosciences, Lithuanian University of Health Sciences, Eiveniu str. 4, LT-50161 Kaunas, Lithuania; 4 Institute of Cardiology, Lithuanian University of Health Sciences, Sukileliu 17, LT-50009 Kaunas, Lithuania; 5 Department of Biochemistry, Medical Academy, Lithuanian University of Health Sciences, Eiveniu str. 4, LT-50161 Kaunas, Lithuania; 6 Department of Surgery and Institute for Research of Digestive System, Lithuanian University of Health Sciences, Eiveniu str.2, LT-50009 Kaunas, Lithuania

**Keywords:** Global brain ischemia, Microcirculation, Mitochondria

## Abstract

**Background:**

Ischemic brain injury due to stroke and/or cardiac arrest is a major health issue in modern society requiring urgent development of new effective therapies. The aim of this study was to evaluate mitochondrial, microcirculatory, and histological changes in a swine model of global cerebral ischemia.

**Results:**

In our model, significant microcirculatory changes, but only negligible histological cell alterations, were observed 3 h after bilateral carotid occlusion, and were more pronounced if the vascular occlusion was combined with systemic hypotension. Analysis of mitochondrial function showed that LEAK respiration (measured in the presence of pyruvate + malate but without ADP) was not affected in any model of global cerebral ischemia in pigs. The OXPHOS capacity with pyruvate + malate as substrates decreased compared with the control levels after bilateral carotid artery occlusion, and bilateral carotid artery occlusion + hypotension by 20% and 79%, respectively, resulting in decreases in the respiratory control index of 14% and 73%, respectively. OXPHOS capacity with succinate as a substrate remained constant after unilateral carotid artery occlusion or bilateral carotid artery occlusion, but decreased by 53% after bilateral carotid artery occlusion and hypotension compared with controls (p < 0.05, n = 3–6). Addition of exogenous cytochrome c to mitochondria isolated from ischemia brains had no effect on respiration in all models used in this study.

**Conclusions:**

We found a decrease in microcirculation and mitochondrial oxidative phosphorylation activity, but insignificant neuronal death, after 3 h ischemia in all our pig models of global cerebral ischemia. Dysfunction of the mitochondrial oxidative phosphorylation system, particularly damage to complex I of the respiratory chain, may be the primary target of the ischemic insult, and occurs before signs of neuronal death can be detected.

## Background

Post cardiac arrest brain injury is a common cause of morbidity and mortality [1]. Although ischemic stroke is the third most common cause of death in the United States and Europe, the only currently approved medical treatment is the administration of intravenous recombinant tissue plasminogen activator within 4.5 hours of stroke onset (according to the European Stroke Organisation guidelines), with the aim of restoring cerebral blood flow. Even if patients survive the acute episode of cerebral ischemia, approximately 15–30% of patients remain disabled at 3 months after onset of stroke, and approximately 20% require permanent medical care in nursing and supportive care institutions [2-8]. These major health issues require urgent development of novel and more effective therapies.

There are a number of experimental models of global cerebral ischemia used for pre-clinical studies of pharmacological interventions, including two or four vessel occlusion or cardiac arrest induction [9-11]. In larger animals such as dogs and pigs, it is easier to perform physiological monitoring and measurements of cerebral blood flow and metabolism. Furthermore, the brains of large animals are gyrencephalic, as for humans. However, the presence of the *rete mirabile* at the base of the brain makes it difficult to create cerebral infarction in pigs [9,12]. A number of studies have suggested that occlusion of both common carotid arteries combined with induction of hypotension or hypoxia for a limited time can induce brain ischemia similar to that observed during cardiac arrest and/or massive ischemic stroke [11]. However, such studies of brain ischemia in pigs provide little or no data on the exact location and severity of brain gray matter injury. Furthermore, there are no studies demonstrating alterations of microcirculation using direct Sidestream Dark Field (SDF) imaging or changes of mitochondrial respiration in the brain of these experimental models.

Thus, in the present study we created a global brain ischemia model in pigs and evaluated acute molecular, microcirculatory and histological changes.

## Methods

### Animals

Animals were treated following guidelines for the care and use of experimental animals of our institution in accordance to applicable laws. The study protocol was approved by the Lithuanian Animal Ethics Committee (SFVS Permission number 0204).

### Anesthesia and surgical preparation

Seventeen 10–12-week-old female Lithuanian White pigs were fasted for 12 h before experimentation, with free access to water. Anesthesia was initiated by intramuscular injection of ketamine (20 mg/kg), xylasine (2 mg/kg) and atropine (0.01 mg/kg), completed by ear vein injection of sodium thiopental (6 mg/kg). After endotracheal intubation, pigs were ventilated using a volume-controlled mode (Drager, Lubeck, Germany) under the following conditions: fraction of inspired oxygen (FiO2) of 0.21 at 14–16 breaths/min and tidal volume of 10 mL/kg to maintain normocapnia.

Anesthesia was maintained by continuous infusion of sodium thiopental (5 mg/kg/h) and fentanil (0.01 mg/kg/h). Paralysis was achieved with intravenous pipecuronium bromide boluses as required. Ringer’s solution (10 mL/kg/h) was administered continuously. A standard lead II electrocardiogram (ECG) was used to monitor cardiac rhythm. To ensure an appropriate depth of anesthesia, we monitored indirect measurements such as tail-clamping, monitoring of the corneal reflex, and lacrimation, as well as changes in hemodynamics and heart rate.

A saline-filled central venous catheter (7-French) was inserted in the right or left femoral vein for drug administration. Core body temperature was monitored continuously via the esophageal temperature probe and kept at >38.0°C using warmed solutions and heating mattresses. An arterial line was placed into the left or right femoral artery to measure invasive arterial blood pressure and to obtain blood gases.

Depending on the group, the neck area was surgically opened to expose the internal carotid arteries bilaterally or unilaterally, and after placing a monofilament nylon hook around one or both arteries, the wound was closed. A standard craniotomy was performed in the temporoparietal region, avoiding injury to the medial venous sinus, to perform direct SDF imaging and to obtain tissue samples for assessment of mitochondrial function, histology, and apoptosis. A thorough hemostasis was achieved prior to the microcirculation measurements and tissue harvesting using monopolar coagulation and bone wax.

### Experimental groups

Immediately after intubation, all animals were randomized to either of four groups: control (C), unilateral carotid occlusion (UCO), bilateral carotid occlusion (BCO), and bilateral carotid occlusion with systemic hypotension (BCOH). In the UCO group a single side carotid artery ligation was performed. After 3 h of cerebral ischemia, a craniotomy was performed for microcirculatory evaluation and tissue sampling for further analysis. Tissue samples of the brain were immediately immersed into formaldehyde (for histological studies), buffer solution (mitochondrial studies), or snap-frozen in liquid nitrogen (stored at -80°C) for cytokine analysis. The BCO group received bilateral carotid artery ligation and craniotomy at 3 h after induction of ischemia. In the BCOH group the common carotid arteries were bilaterally occluded and blood was withdrawn from the arterial line into a heparinized syringe to reduce mean arterial pressure (MAP) to 40–50 mm Hg. Arterial hypotension was maintained at this level over 3 h by continuous MAP titration (e.g., appropriate blood infusion/withdrawal), followed by craniotomy. In the control group, craniotomy was performed after 3 h. After brain preparation the animals were euthanatized by an overdose of sodium thiopental and potassium chloride.

### Hemodynamic and blood gas measurements

Heart rate, mean arterial pressure, temperature, and saturation were continuously recorded. Arterial blood samples were obtained before common artery ligation in stabilized animals and at 3 h after ligation for measurement of hemoglobin, hematocrit, glucose, potassium, arterial lactate, and arterial blood gases (ABL 500; Radiometer, Copenhagen, Denmark).

### Microcirculatory evaluation

Images of the cerebral cortex microcirculation were obtained with side dark field (SDF) video microscopy (Microscan®, Microvision Medical, Amsterdam, Netherlands). SDF imaging device after dura opening was gently applied to the center of the frontal lobe of the affected side corresponding to three to five different innermost parts of approximately 10 × 6 cm in area, and avoiding pressure artifacts. Sequences of 20 s from at least three areas were recorded on a hard disk using a personal computer and AVA v3.0 software (Microvision Medical). Video clips were blindly analyzed offline by two investigators in random order to prevent coupling. Assessment of microvascular flow index (MFI) was performed as previously reported [13]. Vessels were separated into small (mostly capillaries) with a diameter below 20 μm, medium with a diameter 20–50 μm, and large with an inner diameter >50 μm. The final MFI score was the average values of 3–5 × 4 quadrants.

### Assessment of mitochondrial function

#### Preparation of brain mitochondria

Brain tissue from the frontal and frontotemporal areas was cut and homogenized in a glass-Teflon homogenizer in the isolation buffer (10 mL/g of tissue) containing 220 mM mannitol, 75 mM sucrose, 5 mM HEPES, and 1 mM EGTA, pH 7.4 at 4°C. Mitochondria were separated by differential centrifugation (4 min × 1000 g, 10 min × 12000 g). The pellet was resuspended in the same buffer to approximately 50 mg/mL protein and stored on ice. Total mitochondrial protein was measured by a modified Biuret method [14].

#### Measurements of mitochondrial respiratory rates (by high-resolution respirometry)

Respiration was measured using Oxygraph-2k (OROBOROS Instruments, Innsbruck, Austria) at 37°C in 2 mL of medium containing 110 mM KCl, 0.45 M MgCl_2_, 5 mM KH_2_PO_4_, and 10 mM Tris–HCl, pH 7.2 [15]. Datlab software (OROBOROS Instruments) was used for data acquisition and analysis. LEAK respiration was measured in the presence of pyruvate + malate (PM) (6 mM + 6 mM) without ADP. OXPHOS capacity was measured as the rate of oxygen consumption coupled to phosphorylation of ADP to ATP after addition of saturating ADP concentration (2 mM). Complex I was inhibited by 2 mM amytal to measure ETS capacity with electron input through complex II only after addition of 10 mM succinate (S). Atractyloside (0.1 mM) was added to measure the conductance of inner mitochondrial membrane. To test the intactness of the outer mitochondrial membrane, the cytochrome c test was used, for which exogenous cytochrome c (32 μM) was added. Mitochondrial respiration rates were expressed as pmolO_2_/s/0.25 mg protein. The final mitochondrial protein concentration in all experiments was 0.25 mg/mL.

### Histological analysis

At the end of each experiment, the brain tissue from the frontal and frontotemporal areas was removed and immersed in 4% paraformaldehyde for 7 days, and then embedded in paraffin. Paraffin sections were prepared and stained with Cresyl violet and hematoxylin and eosin (H&E). The stained sections were examined by an independent examiner blinded for the experimental protocol and the quantitative analysis of morphological alterations was performed. The number of neurons was estimated in 0.3–0.5 μm sections of the brain preparations (in a series of random samples representing each experimental group) by exclusively counting the nerve cells containing clearly-visible nuclei stained by H&E. The density of the neurons was estimated as the number of the neurons per section area, expressed as N/100.000 μm^2^.

### TUNEL assay

Brain tissue from the frontal and frontotemporal areas was evaluated with an in situ apoptosis detection kit (NeuroTACS™ II; R&D Systems, Minneapolis, MN, USA) as recommended by the manufacturer. Brain sections from the control, UCO, BCO, and BCOH animals were examined and the number of apoptotic cells was calculated under a light microscope. TUNEL positive cells were counted in three separate fields for each animal.

### Statistical analysis

Each group consisted of 4–8 animals. Data are expressed as mean ± standard error of the mean (SEM). Statistical significance was analyzed by analysis of variance (ANOVA) and the Student’s t-test, or the Kruskal-Wallis test and the Mann–Whitney U-test. Significance was accepted at p < 0.05. The statistical package SPSS v.17 (SPSS Inc., Chicago, IL, USA) was used for data analysis.

## Results

Experimental evaluations were performed in a total of 17 pigs. Systemic hemodynamic and metabolic variables in the groups are presented in Table 1. There were no significant differences between the groups in terms of hemodynamic and metabolic variables at baseline (before common artery ligation). A significant decrease in MAP by approximately 50% and an increase in HR by approximately 25% were observed in the BCOH group when compared with baseline (before carotid ligation) and the control group (Table 1). A small but significant decrease in arterial pH was also observed in the BCOH group compared with baseline and the control group. During microcirculatory evaluation, perfusion of the capillaries was fully stopped in the frontal cortex after bilateral carotid artery occlusion, while flow in the larger vessels was still detectable (Table 2).

**Table 1:** Physiological variables

	**Group**			
	**Control (n = 6)**	**UCO (n = 3)**	**BCO (n = 4)**	**BCOH (n = 4)**
Body weight (kg)	19.5 ± 2.8	17.3 ± 2.3	22.8 ± 3.3	22.0 ± 3.4
HR (beats/min)				
Before carotid ligation	125 ± 15	112 ± 11	122 ± 21	112 ± 25
At brain biopsy	111 ± 15	115 ± 9	103 ± 15	138 ± 16^ab^
MAP (mmHg)				
Before carotid ligation	101 ± 14	92 ± 4	105 ± 6	99 ± 17
At brain biopsy	94 ± 11	85 ± 9	89 ± 18	42 ± 7^ab^
Arterial pH (mmHg)				
Before carotid ligation	7.50 ± 0.01	7.40 ± 0.01	7.53 ± 0.03	7.49 ± 0.06
At brain biopsy	7.49 ± 0.02	7.52 ± 0.02	7.55 ± 0.04	7.39 ± 0.01^ab^
Arterial pO2 (mmHg)				
Before carotid ligation	107.2 ± 27.1	116.0 ± 48.1	98.3 ± 30.1	76.8 ± 11.6
At brain biopsy	100.0 ± 15.0	120.0 ± 50.5	82.7 ± 7.3	71.7 ± 9.8
Arterial pCO2 (mmHg)				
Before carotid ligation	38.8 ± 6.1	38.3 ± 7.6	41.0 ± 6.5	38.1 ± 6.3
At brain biopsy	35.0 ± 8.0	37.0 ± 1.4	36.3 ± 4.6	41.3 ± 7.0
Esophageal temperature (ºC)				
Before carotid ligation	39.3 ± 1.0	39.5 ± 1.0	38.3 ± 0.6	38.6 ± 0.9
At brain biopsy	39.4 ± 0.9	39.0 ± 1.2	38.0 ± 0.7	37.8 ± 0.7

BCO, bilateral carotid occlusion; BCOH, bilateral carotid occlusion with systemic hypotension; MAP, mean arterial blood pressure; UCO, unilateral carotid occlusion. Values are mean ± SD. ^a^p < 0.05 *vs.* before carotid ligation; ^b^p < 0.05 *vs.* control.

**Table 2:** Microvascular flow index (MFI)

	**Control**	**UCO**	**BCO**	**BCOH**
MFI of small vessels (<20 μm)	2.75 ± 0.20	1.13 ± 0.21	0	0
MFI of medium vessels (21–50 μm)	3.00 ± 0.00	2.63 ± 0.62	2.08 ± 1.39	1.10 ± 0.45
MFI of large vessels (51–100 μm)	3.00 ± 0.00	2.00 ± 1.18	2.20 ± 1.46	2.05 ± 1.4

BCO, bilateral carotid occlusion; BCOH, bilateral carotid occlusion with systemic hypotension; UCO, unilateral carotid occlusion. Values are mean ± SD.

**Figure 1:**
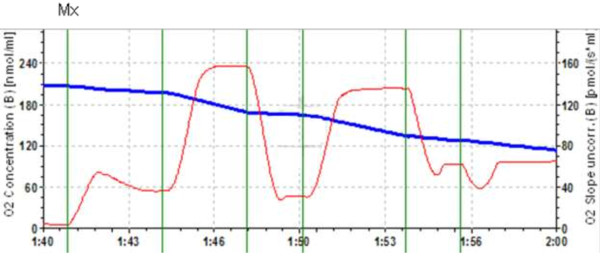
**A typical trace of respirometric measurements of mitochondria isolated from brain of normal piglets.** Substrates were 6 mM pyruvate + 6 mM malate. Further additions: MX - 0.25 mg of mitochondria; ADP - 2 mM ADP; Amit - 2 mM amytal; Succ – 10 mM succinate; ATR – 0.1 mM atractyloside; Cyt c - 32 μM cytochrome c. The blue trace indicates oxygen concentration, the red trace – oxygen flux.

To investigate the effect of cerebral ischemia *in vivo* induced by unilateral and bilateral carotid artery occlusion, with or without hypotension, on mitochondrial parameters in pigs, we monitored their respiration rate in various metabolic states. A typical experimental design for measurement of mitochondrial respiratory functions is presented in Figure 1. At the start of the experiment, the basal (LEAK) respiration rate with complex I dependent substrates pyruvate + malate was measured. OXPHOS capacity was recorded after addition of ADP, and then complex I was inhibited by addition of amytal and succinate. The intactness of the mitochondrial outer membrane was then tested by addition of atractyloside and cytochrome c.

**Figure 2:**
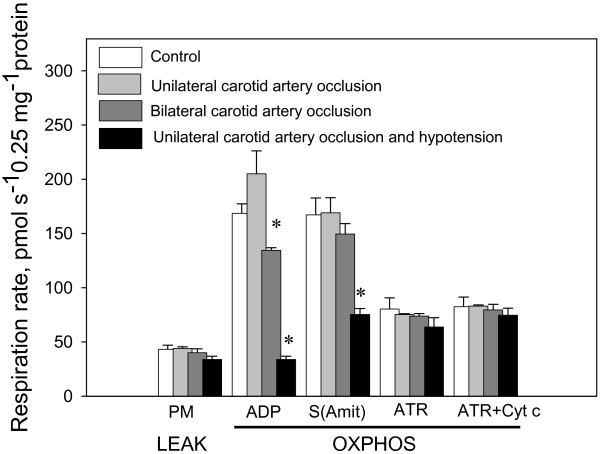
**The effect of different models of global cerebral ischemia in piglets on the mitochondrial respiratory parameters (substrate, pyruvate + malate or succinate).** Supplements were added to mitochondria in the following order: 6 mM pyruvate + 6 mM malate; 2 mM ADP; 10 mM succinate + 2 mM amytal; 0.1 mM atractyloside; 32 μM cytochrome c. p<0.05, n=3-6.

LEAK respiration (which was measured in the presence of pyruvate + malate, but without ADP), was not affected by ischemia in any model (Figure 2). OXPHOS capacity with pyruvate + malate as substrates decreased by 20% and 79% after bilateral carotid artery occlusion and bilateral carotid artery occlusion and hypotension, respectively, compared with the control level, resulting in a decrease of RCI (ADP/PM) by 14% and 73%, respectively. OXPHOS capacity with succinate as the substrate remained constant after unilateral carotid artery occlusion or bilateral carotid artery occlusion, but decreased by 53% after bilateral carotid artery occlusion and hypotension compared with the control level. Mitochondrial respiration rates after addition of atractyloside and cytochrome c were the same in all experimental groups, suggesting that the intactness of the mitochondrial outer membrane was not affected by cerebral ischemia.

**Figure 3:**
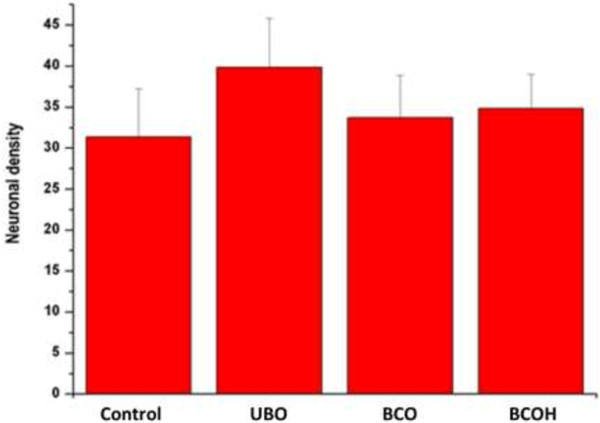
**Neuronal density in different models of brain ischemia.** The graph demonstrates the mean number ± standard error of the neuronal density within the groups: control (Control), unilateral (UCO), bilateral ligation (BCO) of the carotid arteries, bilateral ligation and controlled hypotension (BCOH). The differences between the control and all experimental groups were statistically insignificant at p < 0.05.

Quantitative histological analysis did not reveal any statistically significant differences between the means of neuronal density in various study groups (Figure 3). Neuronal density was 31 ± 6 (range 4–76) in the Control group, 40±6 (range 21–62) in the UCO group, 34±5 (range 8–50) in the BCO group, and 35±4 (range 24–47) in the BCOH group. The percentage of neuronal density ranged from 22–28% in all groups. The differences between the control and all experimental groups were not significant.

**Figure 4:**
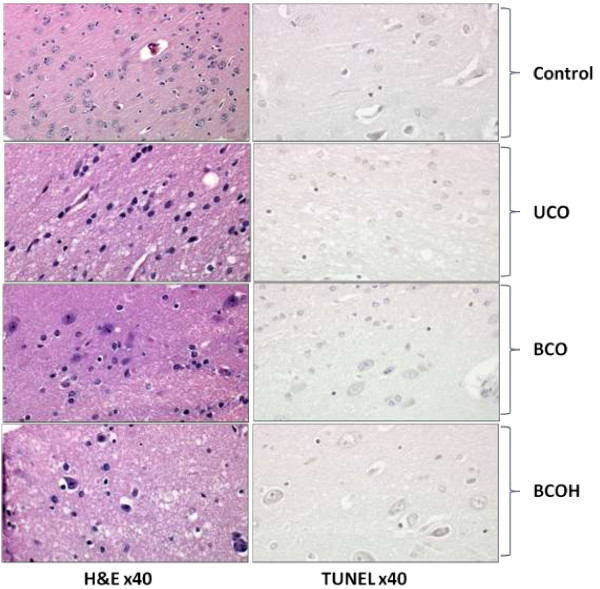
**Morphological changes of tissue in different models of brain ischemia.** The stained sections were examined by independent examiner with a light microscope without the examiner knowing the experimental protocol. Analysis revealed that there are little or none histological changes in the study groups: normal control (Control), animals subjected to the unilateral (UCO), bilateral ligation (BCO) or bilateral ligation of the carotid arteries and controlled hypotension (BCOH). TUNEL assay showed no signs of apoptosis and DNA fragmentation in either of the experimental groups, confirming that many of the cells remain completely intact during short periods of ischemia at least before the reperfusion injury occurs.

TUNEL assay (Figure 4) showed no signs of apoptosis and DNA fragmentation in either of the experimental groups, confirming that the majority of cells remained intact during short periods of ischemia, prior to reperfusion injury.

## Discussion

In this study, we performed a detailed and complex investigation of changes in mitochondrial respiration, microcirculation, and histology during global cerebral ischemia in the pig. The use of appropriate animal models of cerebral ischemia is essential for furthering our understanding of the mechanisms of ischemic brain injury. Several models of cerebral ischemia have been developed in different species [9-11,16-18]. Model of cerebral ischemia should mimic the pathophysiological changes found in human stroke, use procedures that are relatively simple and non-invasive, of low financial cost, and enable monitoring of physiologic parameters and analysis of brain tissue for outcome measures. Although the pig model has a high cost and is labor intensive, the larger animal size makes it easier perform physiological monitoring at multiple time points in the same animal. The technique of induced ventricular fibrillation for establishing global cerebral ischemia is commonly performed in large animals [17,19], and is very similar to the physiological changes occur during human cardiac arrest. Although the use and development of primates and higher mammalian stroke models is an important future goal, small animal models remain widely used at the preclinical level [10,20,21].

The three models of cerebral ischemia used in the present study involved unilateral and bilateral carotid artery occlusion, and bilateral occlusion with hypotension. These surgical procedures produced different effects on microcirculation, with unilateral occlusion for 3 h resulting in a 50% reduction of flow through the small capillaries in the frontal cortex, while 3 h bilateral carotid artery occlusion (without or with hypotension) completely blocked perfusion of these capillaries. Perfusion of medium blood vessels in the frontal cortex was less affected in these models of cerebral ischemia, and flow through large vessels remained constant. Tissue sampling for mitochondrial and histological evaluation was performed in the same region of the cortex after direct visualization of the cortical surface. We chose a 3 h ischemic period because thrombolytic therapy, leading to restoration of blood flow to the ischemic area, could be performed during the first 3–4.5 h after onset of ischemia [22]. Therefore, it is important to explore the processes occurring in the brain during that time.

The impairment of capillary blood flow after carotid occlusion is an expected finding, and may cause mitochondrial dysfunction despite the presence of the *rete mirabile.* Using the same technique, Perez-Barcena et al. [23] found significant microvascular blood flow alterations in small, medium, and large vessels in humans who had undergone decompressive surgery as a result of a space-occupying hemispheric infarction when compared with nonstroke control patients. In our study, the relatively high blood flow in medium and large vessels in the UCO group may represent better protective circulation in pigs.

We also observed a decrease in mitochondrial oxidative phosphorylation, but no neuronal death, after 3 h ischemia in all models used. This suggests that dysfunction of mitochondrial oxidative phosphorylation system, and damage to complex I of the respiratory chain in particular (reflected in the decreased respiration with pyruvate but not succinate), may be the primary target of an ischemic insult, and occurs before signs of neuronal death can be detected (by histological methods and TUNEL staining), in our pig model of global cerebral ischemia. Thus, we established that bilateral carotid occlusion together with systemic hypotension is a reliable method to cause cerebral ischemia in pigs without major disruption of brain tissue morphology, and that disruption of mitochondrial respiratory function may be an indicator of early brain tissue damage.

Morphological changes assessed by histology are traditionally used to identify dead or dying cells. Visualization of cell death after ischemic stroke by light microscopy can be used to visualize and measure the complete area of infarction or to analyze alterations at the single cell level. However, a major challenge of this technique is reliable detection of early ischemic changes. Nevertheless, numerous staining methods have been developed to overcome these limitations, including routine histology, silver staining, and fluorescence markers. It is important to note that each of the methods has advantages and disadvantages and there is no 'best’ solution. The most frequently used routine histological stains include hematoxylin and various modifications of the Nissl stain, which can be quickly and easily performed on tissue sections. Using these standard protocols, numerous studies have described the distinct temporal and spatial patterns of postischemic morphological alterations [24]. However, because the progression of postischemic changes depends on the severity and duration of ischemia, the pathology observed can only be reliably interpreted under their specific standardized experimental conditions. In general, there is a loss of Nissl substance detectable approximately 2–3 h after experimental ischemia. Our study confirmed that such morphological changes are not pronounced and are difficult to evaluate objectively in the early period of experimental brain ischemia. These limitations of routine staining procedures can be overcome by the use of suppressed silver stains, fluorescence-based techniques, or evaluation of other parameters such as microcirculation and/or mitochondrial function.

It is now well documented that mitochondria play a central role in the pathophysiology of many neurodegenerative diseases, including stroke and ischemic brain injury. Several potentially deleterious mitochondrial responses including impaired ability to generate ATP [25], induction of free radical production and formation of a mitochondrial permeability transition pores (mPTPs) [26], and membrane permeabilization resulting in release of factors that promote apoptotic cell death [27,28], have been detected during cerebral ischemia. Measurement of the respiratory activity of isolated mitochondria is a sensitive method to determine persistent alterations in mitochondrial function induced by ischemia or other insults. Using this method, we determined that the primary cerebral ischemia-induced injury to mitochondria involved a decrease in phosphorylating respiration (or OXPHOS capacity) with NADH-dependent substrates at 3 h of bilateral carotid artery occlusion, which developed further when bilateral artery occlusion was combined with hypotension. OXPHOS capacity with succinate as a substrate was inhibited only for bilateral artery occlusion with hypotension. These data suggest that suppression of complex I of the mitochondrial respiratory chain was likely the earliest event in the development of ischemic injury in cerebral mitochondria during ischemia. However, we cannot exclude ischemia-induced inactivation or degradation of the pyruvate dehydrogenase complex [29], which may also lead to inhibition of mitochondrial respiration with NAD-linked substrates. In support of these results, it was previously reported that brain mitochondrial respiration supported by either NAD-linked or FAD-linked substrates exhibited similar changes in rats [30], while the activity of the complexes I, II, and III of the mitochondrial respiratory chain were decreased after global ischemia in the rat brain [30-32]. Furthermore, brain ischemia does not affect complex IV activity, although a long period of reperfusion markedly inhibited its activity [30]. In our pig models, ischemia-induced inhibition of respiration with both substrates was not induced in the presence of exogenous cytochrome c, suggesting that this inhibition was not caused by loss of cytochrome c from brain mitochondria. This finding is in contrast to observations that loss of cytochrome c in heart mitochondria was the earliest event during ischemic injury, and occurred well before inhibition of complex I activity [33]. We also found that LEAK respiration (with pyruvate + malate) was not affected by ischemia in any experimental group, suggesting that proton permeability of the mitochondrial inner membrane was not changed. This also contrasts with results from heart mitochondria where ischemia has been shown to cause an increase in proton leak [33]. Therefore, our study suggests that there are differences in the dynamics of ischemic injury development in mitochondria between pig and rat models of cerebral ischemia. There are also differences in comparison with ischemic damage to heart mitochondria. Furthermore, our data suggest that mitochondria play a key role in global cerebral ischemia, and may be a potential target for neuroprotection.

## Conclusions

Our three experimental models of cerebral ischemia in pigs are reliable and useful tools to investigate the mechanism of cerebral ischemia and neuroprotection. Simultaneous use of histological, mitochondrial respiration and microcirculatory evaluation techniques allows detailed study of the mechanisms involved in the development of cerebral ischemia. Along with decreased microcirculation, we observed a decrease in the activity of mitochondrial oxidative phosphorylation, but insignificant neuronal death, after 3 h ischemia in all models used. Dysfunction of the mitochondrial oxidative phosphorylation system, particularly damage to complex I of the respiratory chain, may be the primary target of the ischemic insult and occurs before signs of neuronal death can be detected in our pig model of global cerebral ischemia.

## Competing interests

The authors declared that they have no competing interests.

## Authors’ contributions

OS, ZD, AP, DV, and VB initiated the study, developed the study protocol, analyzed and interpreted the data, and wrote the manuscript. OS, ZD, and AP performed the experiments, and analyzed and interpreted the data. DV and VV coordinated the logistics of the study. GB performed mitochondrial respirations measurements. OS, AP, GB, and ZD performed the statistical analyses. All authors drafted the manuscript and revised it critically for important intellectual content and approved the final manuscript.
